# Consolidative allogeneic hematopoietic stem cell transplantation after chimeric antigen receptor T-cell therapy for relapsed/refractory B-cell acute lymphoblastic leukemia: who? When? Why?

**DOI:** 10.1186/s40364-020-00247-8

**Published:** 2020-11-25

**Authors:** Huiwen Jiang, Yu Hu, Heng Mei

**Affiliations:** 1 Institute of Hematology, Union Hospital, Tongji Medical College, Huazhong University of Science and Technology, Wuhan, 430022 China; 2 Hubei Clinical Medical Center of Cell Therapy for Neoplastic Disease, Wuhan, 430022 China

**Keywords:** Chimeric antigen receptor-modified T-cell therapy, Allogeneic hematopoietic stem cell transplantation, Relapsed or refractory B-cell acute lymphoblastic leukemia, Prognosis

## Abstract

Although anti-CD19 chimeric antigen receptor (CAR) T-cell therapy shows good efficacy in patients with relapsed/refractory B-cell acute lymphoblastic leukemia (r/r B-ALL), it fails to improve long-term leukemia-free survival (LFS). Allogeneic hematopoietic stem cell transplantation (allo-HSCT) after CAR T-cell therapy has emerged as a promising strategy to prolong LFS. Nevertheless, which patients are likely to benefit from consolidative allo-HSCT, as well as the optimal therapeutic window, remain to be explored. Recent clinical data indicate that patients with complex karyotypes, adverse genes, and high pre-infusion minimal residual disease (MRD) by flow cytometry in the bone marrow, were at high risk of relapse after CAR T-cell therapy. High pre-lymphodepletion lactate dehydrogenase, low pre-lymphodepletion platelet count, absence of fludarabine in lymphodepletion, persistent leukemic sequence by high throughput sequencing in bone marrow after CAR T-cell infusion, and early loss of CAR T cells have also been linked to relapse after CAR T-cell therapy. In patients having these risk factors, consolidative allo-HSCT after CAR T-cell therapy may prolong LFS. Allo-HSCT provides optimal clinical benefit in patients with MRD-negative complete remission, typically within three months after CAR T-cell therapy. Herein, we summarize the clinical data on consolidative allo-HSCT after anti-CD19 CAR T-cell therapy, as well as the potential factors associated with allo-HSCT benefit. We also discuss the optimal therapeutic window and regimen of consolidative allo-HSCT. Finally, and most importantly, we provide recommendations for the assessment and management of r/r B-ALL patients undergoing anti-CD19 CAR T-cell therapy.

## Background

Patients with relapsed/refractory B-cell acute lymphoblastic leukemia (r/r B-ALL) often have clinicopathological characteristics associated with poor prognosis, such as high tumor burden and high-risk gene mutations. Conventional therapies typically fail to achieve minimal residual disease (MRD)-negative complete remission (CR). However, advances in chimeric antigen receptor (CAR) T-cell therapy have dramatically improved the treatment of r/r B-ALL [1–3] The first FDA-approved CD19-targeted CAR T-cell therapy, Tisagenlecleucel, has recently been established as the standard of care for r/r B-ALL patients aged under 26 as per the NCCN Guidelines (https://www.nccn.org/) [4–6]. However, despite the unprecedented remission rate and controllable side effects, disease relapse after CAR T-cell therapy remains a significant challenge [7, 8]. Early loss or exhaustion of CAR T cells, selection of CD19-negative clones, downregulation of CD19 expression, lineage switch of leukemia, and tumor microenvironment are important factors contributing to relapse after CAR T-cell therapy [9, 10]. Hence, the development of new therapeutic strategies that overcome these obstacles and achieve durable remission in r/r B-ALL patients are urgently warranted.

For decades, allogeneic hematopoietic stem cell transplantation (allo-HSCT) has been regarded as the only well-established curative cellular therapy for patients with B-ALL. However, r/r B-ALL patients with MRD cannot achieve a satisfactory response and have a higher relapse rate after allo-HSCT than patients without MRD [11–17]. Furthermore, r/r B-ALL patients may develop severe organ damage or infection after aggressive chemotherapy and, hence, be precluded from allo-HSCT. For these r/r B-ALL patients with MRD, CAR T-cell therapy can serve as an effective and safe method to induce MRD-negative CR before subsequent allo-HSCT [18–25]. The combination of CAR T-cell therapy and allo-HSCT has been suggested to reduce the relapse rate of leukemia. Notably, several studies found that patients receiving consolidative allo-HSCT have longer leukemia-free survival (LFS) than the patients receiving CAR T-cell therapy alone [26–28]. Nonetheless, consolidative allo-HSCT is not recommended for all patients because it will increase the economic burden and bring risk of severe toxicity, such as graft-versus-host disease (GVHD). Besides, when and how to perform consolidative allo-HSCT are still not well defined. Therefore, clinicians should perform thorough benefit and risk assessment before using consolidative allo-HSCT in r/r B-ALL patients after CAR T-cell therapy.

Herein, we review the findings from large-scale clinical trials of CAR T-cell therapy and consolidative allo-HSCT and summarize the potential factors associated with consolidative allo-HSCT benefit. Finally, we provide a recommendation to evaluate and transfer r/r B-ALL patients to consolidative allo-HSCT.

## Anti-CD19 CAR T-cell therapy and consolidative Allo-HSCT for r/r B-ALL

In a number of clinical trials, some r/r B-ALL patients received consolidative allo-HSCT after anti-CD19 CAR T-cell therapy. The patient characteristics, costimulatory domains of CAR T cells, lymphodepleting chemotherapies before CAR T-cell infusion, subsets and doses of infused CAR T cells, CR rates and duration after CAR T-cell therapy, and consolidative allo-HSCT application methods are summarized in Table 1.

**Table 1:** Clinical trials of anti-CD19 CAR T-cell therapy and consolidative allo-HSCT for r/r B-ALL

Register number	Population (treated/enrolled) and age	Prior HSCT	Costimulatory domain and transduction method	Lymphodepletion	Subset and dose of infused CAR T cells	ORR^**b**^ and MRD^**−**^ CR rate	Long-term survival	Patient number and time for consolidative allo-HSCT	Reasons for not receiving allo-HSCT	transplant-related toxicities	Relapse rate, % (n)	Relapse rate after consolidative HSCT	Relapse rate without consolidative HSCT
NCT01593696 [21]	20/20 5 to 27	35.0% (7/20)	CD28, retroviral	Flu/Cy	unselected, 1 × 10^6^/kg to 3 × 10^6^/kg	70.0% (14/20), 60.0% (12/20)	OS: 51.6% after 9.7 m, EFS: 78.8% after 4.8 m	Patient number: 83.3% (10/12) MRD^−^ CR patients, time: 45 to 82 (median 51) days	ineligible	no unexpected toxicity	16.7% (2/12) MRD^−^ CR patients	0.0%, (0/10)	100.0% (2/2)
ChiCTR-llh-16008711 [26]	51/51 2 to 68	/	4-1BB, lentiviral	Flu/Cy	unselected, 0.05 × 10^5^/kg to 14 × 10^5^/kg	91.8% (45/49), 87.8% (43/49)	OS: /, LFS: 81.3% (6 m) after HSCT	Patient number: 60.0% (27/45) CR/CRi patients, time: 35 to 293 (median 84) days	/	/	24.4% (11/45) CR/CRi patients	7.4% (2/27)	50.0% (9/18)
NCT01626495 & NCT01029366 [20]	30/30^a^ 5 to 60	60% (18/30)	4-1BB, lentiviral	multiple	unselected, 0.76 × 10^6^/kg to 20.6 × 10^6^/kg	90% (27/30), 73.3% (22/30)	OS: 78% (6 m), EFS: 67% (6 m)	Patient number: 11.1% (3/27) CR patients, time: /	lack of suitable donor, prior HSCT, family choice	/	25.9% (7/27) CR patients	0.0% (0/3)	29.2% (7/24)
NCT02435849 [4]	75/92 3 to 23	61.3% (46/75)	4-1BB, lentiviral	Flu/Cy or Ara-C/VP16	unselected, 0.2 × 10^6^/kg to 5.4 × 10^6^/kg	81.3% (61/75), 81.3% (61/75)	OS: 90% (6 m), 76% (12 m), EFS: 73% (6 m), 50% (12 m), LFS: 80% (6 m), 59% (12 m)	Patient number: 13.1% (8/61) CR/CRi patients, time: within 6 months	/	hepatobiliary disorders	36.1% (22/61) CR/CRi patients	0.0% (0/4), 4 others with unknown status	41.5% (22/53)
NCT01044069 [1]	53/83 ≥18	35.8% (19/53)	CD28, retroviral	Flu/Cy or Cy	unselected, 1 × 10^6^ or 3 × 10^6^/kg	83.0% (44/53), 66.7% (32/48)	OS: median 12.9 m, EFS: median 6.1 m	Patient number: 50.0% (16/32) MRD^−^ CR patients, time: 44 to 312 (median 74) days	/	6 died from transplant-related toxic effects	50.0% (16/32) MRD^−^ CR patients	37.5% (6/16)	62.5% (10/16)
NCT02028455 [2]	43/45 1.3–25.3	65.1% (28/43)	4-1BB, lentiviral	Flu/Cy or Cy	CD4^+^ and CD8^+^ CAR T cells (1:1), 0.5 × 10^6^/kg to 10 × 10^6^/kg	93% (40/43), 93% (40/43)	OS: 69.5% (12 m), EFS: 50.8% (12 m)	Patient number: 28% (11/40) MRD^−^ CR patients, time: /	/	/	45.0% (18/40) MRD^−^ CR patients	18.1% (2/11)	55.2% (16/29)
NCT02772198 [29]	20/21, 5 to 48	50.0% (10/20)	CD28, retroviral	Flu/Cy	unselected, 1 × 10^6^/kg	90.0% (18/20), 78.6% (11/14)	OS: 90% (12 m), EFS: 73% (12 m)	Patient number: 77.8% (14/18) CR patients, time: median 68 days	/	/	22.2% (4/18) CR patients	14.3% (2/14)	50.0% (2/4)
NCT01865617 [27]	53/59, 20 to 76	43.4% (23/53)	4-1BB, lentiviral	Flu/Cy or Cy	CD4^+^ and CD8^+^ CAR T cells (1:1), 2 × 10^5^/kg or 2 × 10^6^/kg	84.9% (45/53), 84.9% (45/53)	(for MRD^−^ CR patients) OS: median 20.0 m, EFS: median 7.6 m	Patient number: 40.0% (18/45) MRD^−^ CR patients, time: 44 to 138 (median 70) days	age, the history of a prior transplant, patient preference, comorbidities	invasive fungal infection (aspergillus pneumonia), idiopathic acute respiratory distress syndrome, hepatic failure secondary to adenovirus infection, GVHD	48.9% (22/45) MRD^−^ CR patients	16.7% (3/18)	70.4% (19/27)
NCT02965092 & NCT03366350 [28]	58/60 ≤70	5.2% (3/58)	4-1BB, lentiviral	Flu/Cy	CD4^+^ and CD8^+^ CAR T cells (1:1), 0.89 × 10^6^/kg to 4.01 × 10^6^/kg	87.9% (51/58), 81.0% (47/58)	OS: median 16.1 m, EFS: median 7.3 m	Patient number: 44.7% (21/47) MRD^−^ CR patients, time: 33 to 89 (median 44) days	previous allo-HSCT, contraindications, lack of suitable donors, personal reasons	GVHD, pulmonary infection	38.3% (18/47) MRD^−^ CR patients	9.5% (2/21)	61.5% (16/26)
NCT03173417 [30]	110/115, 2 to 61	14.5% (16/110)	4-1BB or CD28, lentiviral	Flu/Cy	unselected, 1 × 10^5^/kg to 10 × 10^5^/kg	92.7% (102/110), 87.3% (96/110)	OS: 63.9% (12 m), EFS: 57.9% (12 m)	Patient number: 73.5% (75/102) CR patients, time: 36 to 120 (median 63) days	personal preference, performance status, financial issues, and donor availability	GVHD	22.5% (23/102) CR patients	13.3% (10/75)	48.1% (13/27)

^a^ including one patient with T-ALL

^b^ The rate of patients achieving CR or CR with incomplete count recovery.

/: not mentioned.

*Abbreviations*: *allo-HSCT* allogeneic hematopoietic stem cell transplantation, *Ara-C* cytarabine, *CR* complete remission, *Cy* cyclophosphamide, *EFS* event-free survival, *Flu* fludarabine, *GVHD* graft-versus-host disease, *LFS* leukemia-free survival, *MRD* minimal residual disease, *ORR* overall response rate, *OS* overall survival, *VP16* etoposide

KTE-C19 (axicabtagene ciloleucel) safety and efficacy were assessed in a phase 1 clinical trial of 20 pediatric and young adult r/r B-ALL patients at the National Cancer Institute [21]. Infusion of 1 × 10^6^/kg to 3 × 10^6^/kg CAR T cells resulted in CR in 14 patients, while 12 patients were MRD-negative. Among the 12 MRD-negative CR patients, 10 received allo-HSCT at a median of 51 days after CAR T cell infusion; these patients remained leukemia-free. The remaining two patients were ineligible for allo-HSCT and developed CD19-negative relapse, indicating that the combination of CAR T-cell therapy and allo-HSCT improves long-term LFS. In a follow-up study of 51 B-ALL patients and two lymphoma patients, the 32 newly-recruited patients received 1 × 10^6^/kg CAR T cells along with lymphodepletion therapy comprised of fludarabine (flu) and cyclophosphamide (cy), or ifosfamide/etoposide [31]. Twenty-eight of the 53 patients achieved MRD-negative CR, with a median LFS of 18 months. Lymphodepletion with flu/cy significantly prolonged overall survival (OS). Twenty-one of the 28 MRD-negative CR patients received consolidative allo-HSCT at a median of 54 days after CAR T-cell infusion. Patients treated with allo-HSCT exhibited significantly longer LFS (median LFS not reached) than the seven patients that did not receive allo-HSCT (median LFS, 4.9 months). Additionally, researchers observed a shorter persistence of CD28-based KTE-C19 cells than 4-1BB-based CAR T cells, and hypothesized the difference might derive from CAR T cell exhaustion or immunological mechanisms. Albeit the limited persistence (less than 68 days) of CD28-based CAR T cell, it was sufficient to induce MRD-negative CR and served as an effective bridge to allo-HSCT.

Fifty-one r/r B-ALL patients received 0.05 × 10^5^/kg to 14 × 10^5^/kg anti-CD19 CAR T cells at the Lu Daopei Hospital in China, and 20 patients received final-settled 1 × 10^5^/kg CAR T cells. Forty-five patients achieved CR or CR with incomplete count recovery (CRi) [26]. Twenty-seven responding patients received consolidative allo-HSCT at a median of 84 days after CAR T-cell infusion. Twelve of these patients had complex chromosomal aberrations, 13 had adverse gene mutations (e.g., *IKZF1* and *TP53*), and nine had extramedullary diseases (EMD), including central nervous system leukemia, testicular leukemia, diffused EMDs and others. All patients received myeloablative conditioning chemotherapy before transplantation of grafts from haploidentical donors, matched unrelated donors, or matched sibling donors. Of the 27 transplanted patients, 23 maintained MRD-negative CR (median of 133 days after allo-HSCT), two died of treatment-related mortalities (TRM), and two relapsed. The remaining 18 responding patients refused allo-HSCT, and nine of them relapsed. After adjusting for a uniform time point (90 days after CAR T-cell infusion) to eliminate the influence of early relapse, which precludes subsequent allo-HSCT, researchers found a significant difference in LFS between transplanted and non-transplanted patients. Overall, the study firstly indicated that consolidative allo-HSCT further reduced the relapse rate of r/r B-ALL patients with high-risk gene mutations.

In the phase 1/2a clinical trial conducted at the Children’s Hospital of Philadelphia and Hospital of the University of Pennsylvania, 30 r/r ALL patients received 0.76 × 10^6^/kg to 20.6 × 10^6^/kg CTL019 (tisagenlecleucel) cells and 27 achieved CR (22 were MRD-negative) [20]. Three patients received subsequent allo-HSCT and remained in CR for up to 12 months after CTL019 infusion, demonstrating the feasibility of allo-HSCT as consolidation treatment. Moreover, a correlation was observed between sustained remission and prolonged persistence (over three months) of CTL019 and B-cell aplasia (BCA), as the two patients exhibiting loss of CTL019 and recovery of normal B cells subsequently developed CD19-positive relapse. Therefore, researchers suggested that the sign of normal B cell return could potentially provide a window for consolidative allo-HSCT.

Subsequently, a phase 2, single-cohort, multicenter clinical trial of tisagenlecleucel was carried out with 75 r/r B-ALL patients [4]. Infusion of 0.2 × 10^6^/kg to 5.4 × 10^6^/kg CAR T-cells resulted in CR or CRi in 61 patients, all of whom were MRD-negative. The median persistence of tisagenlecleucel in the blood was 168 days at data cut-off, with an ongoing persistence for up to 20 months. All responding patients experienced BCA, and the probability of BCA at six months after CAR T-cell infusion was 83%. Consolidative allo-HSCT was not taken preferentially due to the durable persistence of tisagenlecleucel, which indicated a high probability of cure. Only eight patients underwent allo-HSCT while in remission, two of whom developed MRD-positive disease, and two exhibited B cell recovery within six months of CAR T-cell infusion. All eight transplanted patients remained alive until study endpoint; four of these patients were still in remission. The relapse-free survival (RFS), event-free survival (EFS), and OS were 80, 73, and 90% at six months, and 59, 50, and 76% at 12 months, suggesting the high probability of durable remission after treatment with tisagenlecleucel alone. Therefore, researchers suggested consolidative allo-HSCT only in patients with signs of relapse, such as MRD recurrence and B cell recovery.

In the phase 1 study conducted in Memorial Sloan Kettering Cancer Center, 16 r/r B-ALL patients received 3 × 10^6^/kg 19-28z CAR T cells, resulting in complete molecular remission (CRm) in 12 patients [19]. Two patients refused allo-HSCT, two patients had pre-existing medical contraindications to allo-HSCT, and one patient was under evaluation for allo-HSCT; the remaining 7 CRm patients received consolidative allo-HSCT. At the follow-up of 2 to 24 months, no patients showed relapse after allo-HSCT, although two patients experienced severe transplantation-related complications and eventually died. Overall, the trial showed that 19-28z CAR T-cell therapy provides an effective bridge for patients otherwise either ineligible or eligible but with MRD to receive standard of care allo-HSCT. A follow-up study in 53 patients and with a longer follow-up (median, 29 months) evaluated the efficacy of CAR T cells at a dose of 1 × 10^6^/kg or 3 × 10^6^/kg [1]. Sixteen of the 32 MRD-negative CR patients received allo-HSCT at a median of 74 days after 19-28z CAR T-cell infusion, six of whom relapsed. The remaining 16 MRD-negative CR patients did not receive allo-HSCT, and 10 of them relapsed. No significant difference in OS and EFS was observed between these two groups, indicating that consolidative allo-HSCT did not improve long-term outcomes. It is worth noting that a higher ratio of peak CAR T-cell expansion to baseline tumor burden (rather than the absolute magnitude of T cell expansion or disease burden) was associated with prolonged OS and EFS.

Forty-five pediatric and young adult r/r B-ALL patients were enrolled in a phase 1 clinical trial of SCRI-CAR19v1 conducted in Seattle Children’s Research Institute [2]. The infused CAR T cells comprised of CD4^+^ and CD8^+^ CAR T cells at the ratio of 1:1, and the total dose was 0.5 × 10^6^/kg to 10 × 10^6^/kg. Eleven of the 40 MRD-negative CR patients proceeded to allo-HSCT (two developed CD19-positive relapse), and the remained 29 patients received no consolidative allo-HSCT (16 experienced leukemia recurrence). Researchers found that the early loss of functional CAR T cells reflected by the short duration of BCA (median duration of three months), significantly increased the risk of CD19-positive relapse. Additionally, flu/cy lymphodepletion prolonged the persistence of CAR T cells. Further analysis revealed that functional CAR T cell persistence for more than six months was a critical determinant of durable remission [32]. Furthermore, some T cell-intrinsic features, including elevated expression of LAG3 and reduced secretion of TNF-α in circulating CD8^+^ T cells at the time of leukapheresis, predicted therapeutic failure and early relapse. Therefore, these features can serve as indicators for consolidation therapies. Data from a phase 1/2 trial with a follow-up time of more than one year were further analyzed to evaluate the effect of allo-HSCT on long-term outcomes [23]. Fifty of the 64 enrolled patients achieved CR, 32 of whom received the phase 1 dose and 18 received the phase 2 dose of 1 × 10^6^/kg CAR T cells. Seventeen CR patients had no history of allo-HSCT and exhibited prolonged LFS after treatment with consolidative allo-HSCT following CAR T-cell therapy. However, there was no significant benefit of consolidative allo-HSCT in patients previously treated with allo-HSCT. Interestingly, a certain benefit of consolidative allo-HSCT was observed in CR patients who were at high risk of relapse with an early loss of BCA (before 63 days), regardless of allo-HSCT history. These findings suggest that patients should be considered for consolidative allo-HSCT during remission after CAR T-cell therapy if they have no history of allo-HSCT, or if they had a history of allo-HSCT but with a short persistence of functional CAR T cells.

A phase 1b/2 clinical trial of CD28-based anti-CD19 CAR T-cell therapy was conducted at Sheba Medical Center in Israel [29]. Twenty r/r ALL patients received CAR T-cell infusion (1 × 10^6^/kg). Ten of these patients had a history of allogeneic or haploidentical HSCT, and eight of them had active resistant extramedullary (EM) leukemic involvement at recruitment. The active EM sites included the kidney, spine, femur, temporal lobe, leptomeningeal enhancement, cerebrospinal fluid, paraspinal, scalp, and diffuse bone infiltration. Eighteen patients achieved CR, and 14 of them were referred to consolidative allo-HSCT within a median of 68 days after CAR T-cell infusion. Of these 14 patients, seven had prior transplantation, and five had a history of active EM sites. No severe transplantation-related toxicities were reported. Two of the 14 transplanted patients exhibited CD19-positive relapse, and two of the four non-transplanted patients had CD19-positive and CD19-negative relapse, respectively. The findings of the study demonstrated the safety of consolidative allo-HSCT after CAR T-cell therapy in patients with prior transplantation or EM leukemia and provided preliminary evidence that consolidative allo-HSCT reduces the relapse rate.

In a phase 1/2 clinical trial conducted at the Fred Hutchinson Cancer Research Center, 53 B-ALL patients received 2 × 10^5^/kg or 2 × 10^6^/kg anti-CD19 CAR T cells comprised of CD4^+^ and CD8^+^ cells at a 1:1 ratio [27]. Forty-five patients achieved MRD-negative CR. High throughput sequencing revealed that the absence of the malignant clone in the bone marrow three weeks after CAR T-cell infusion was associated with prolonged EFS in patients with MRD-negative CR. Furthermore, stepwise multivariable modeling demonstrated that low pre-lymphodepletion lactate dehydrogenase (LDH) (≤ 210 U/L), high pre-lymphodepletion platelet count (≥ 100,000/μL), and incorporation of fludarabine into the lymphodepletion regimen were independent prognostic factors of improved EFS in patients with MRD-negative CR. Based on age, history of transplantation, patient preference, and comorbidities, 18 MRD-negative CR patients received consolidative allo-HSCT, and three of these patients experienced CD19-positive relapse. Four patients died due to transplantation-related adverse effects, including invasive fungal infection (aspergillosis), idiopathic acute respiratory distress syndrome, hepatic failure secondary to adenovirus infection, and GVHD. Multivariate analysis revealed that consolidative allo-HSCT was associated with prolonged EFS. Interestingly, a longer time between CAR T-cell infusion and allo-HSCT (≥ 80 days) was associated with a higher risk of death and increased non-relapse mortality [33]. Therefore, researchers concluded that both high-risk and low-risk patients might benefit from allo-HSCT and suggested that eligible adult MRD-negative CR B-ALL patients should undergo allo-HSCT as soon as possible after anti-CD19 CAR T-cell therapy.

In a non-randomized interventional pragmatic clinical trial conducted at our institution, 58 r/r B-ALL patients received 0.89 × 10^6^/kg to 4.01 × 10^6^/kg CAR T cells comprised of CD4^+^ and CD8^+^ T cells at a ratio of 1:1 [28]. Forty-seven of the 58 patients achieved MRD-negative CR. Subsequently, 21 MRD-negative CR patients received consolidative allo-HSCT within three months after CAR T-cell infusion. Patients received peripheral blood stem cell transplantation with or without bone marrow transplantation, with grafts from matched sibling donors or haploidentical donors. Additionally, patients received myeloablative conditioning chemotherapy and cyclosporine/tacrolimus, methotrexate, mycophenolate mofetil, anti-CD25 monoclonal antibody, or anti-thymocyte globulin to prevent GVHD. At a median follow-up time of 10.8 months after CAR T-cell infusion, two patients experienced TRM and two relapsed after consolidative allo-HSCT. Twenty-six MRD-negative CR patients did not receive consolidative allo-HSCT for various reasons, and 16 of them experienced recurrence. EFS and RFS (but not OS) differed significantly between transplanted and non-transplanted patients. Notably, subgroup analyses indicated improved EFS and RFS in patients with high (≥ 5%) pre-infusion bone marrow MRD assessed by flow cytometry, or poor prognostic markers, including complex karyotypes, *BCR/ABL1*, *MLL/AF4*, *TP53*, and *E2A/PBX1* mutations.

In the recent published phase 1/2 clinical trial conducted at the Lu Daopei Hospital in China, 110 pediatric and adult patients received fludarabine and cyclophosphamide lymphodepleting chemotherapy, and single anti-CD19 CAR T-cell infusion of 1 × 10^5^/kg to 10 × 10^5^/kg [30]. Patients were with high-risk factors, including EMDs, *BCR-ABL*, *TEL-AML1*, *E2A-PBX1*, *E2A-HLF* fusion genes, *IKZF1*, *TP53* gene mutations, and post-transplant relapse. Morphologic CR was achieved in 92.7% patients, and MRD-negative CR in 87.3% patients. Of the 102 CR patients, 75 received consolidative allo-HSCT at a median of 63 days (range, 36–120 days) after CAR T-cell infusion. Patients received conventional myeloablative conditioning regimens, and grafts from HLA-identical sibling donors, matched-unrelated donors, or haploidentical donors, and GVHD prophylaxis comprised of cyclosporine, methotrexate, and mycophenolate mofetil. The 1-year OS and LFS were 79.1 and 76.9% for patients bridging into allo-HSCT, and 32 and 11.6% for patients receiving CAR T-cell therapy alone. Multivariable analysis indicated significantly improved OS and LFS in patients with consolidative allo-HSCT. These findings further demonstrated the benefit of consolidative allo-HSCT in patients with pre-treatment high-risk factors.

Taking into consideration that data from the above clinical trials, a total of 429 r/r B-ALL patients achieved CR/CRi after anti-CD19 CAR T-cell therapy. Of these responding patients, 203 proceeded to consolidative allo-HSCT, of whom only 27 (13.3%) relapsed. On the other hand, relapse was observed in 116 (51.3%) of the 226 patients who did not receive consolidative allo-HSCT. Because of differences in CAR design, trial protocol, and patient characteristics, no conclusions can be drawn with confidence; nevertheless, consolidative allo-HSCT seems to reduce the recurrence rate. The time window for consolidative allo-HSCT ranged from 33 days to 312 days after CAR T-cell infusion, and the median time was 44 days to 84 days after CAR T-cell infusion. The most common transplantation-related toxicities were GVHD, infections, and hepatic or respiratory dysfunctions. The main reasons for not providing consolidative allo-HSCT included a suboptimal response to CAR T-cell therapy, age, comorbidities, contraindications, prior HSCT, lack of suitable donor, family choice, and patient preference. Altogether, the findings of these studies suggest that consolidative allo-HSCT may improve long-term outcomes for most r/r B-ALL patients (Table 1).

In addition to the above published studies, there are several clinical trials (NCT03110640, ChiCTR1800017669 … …) ongoing in domestic clinical centers. Despite the bridging strategy is more and more popular among domestic clinical centers, large-scale multicenter clinical trial is still lacked. It is meaningful to conduct a multicenter clinical trial with uniform standards of both CAR T-cell manufacture and patients’ management.

## Factors associated with consolidative Allo-HSCT benefit

By reviewing available data from the literature, we identified several risk factors associated with relapse after CAR T-cell therapy. These factors can be classified into the following categories: (1) pre-treatment (baseline) patient characteristics, such as complex karyotypes or adverse genes, high pre-infusion MRD by flow cytometry in the bone marrow, high pre-lymphodepletion LDH, and low pre-lymphodepletion platelet count; (2) lymphodepletion regimen without fludarabine; (3) post-CAR T-cell infusion parameters, such as persistent leukemic cells in the bone marrow and early loss of CAR T-cells.

### Pre-treatment patient characteristics

Complex karyotypes and overexpression of certain genes, including *MLL/AF4*, *BCR/ABL1*, *E2A/PBX1,* and *TP53*, have been associated with an increased risk of relapse and poor prognosis in r/r B-ALL [34–37]. Numerous studies reported that *MLL*-rearranged, *BCR/ABL1*^+^, and *TCF3-ZNF384*^+^ B-ALL patients were associated with an increased risk of CD19-negative myeloid lineage relapse after CD19-targeted immunotherapy, such as CAR T-cell therapy and blinatumomab [38–41]. The lineage switch can lead to loss of CD19 and resistance to CD19-targeted CAR T-cell therapy. Therefore, patients with chromosomal alterations or high-risk genes should be considered for standard of care allo-HSCT, and CAR T-cell therapy can serve as a suitable bridge to turn related genes negative and transfer patients to allo-HSCT [26, 42]. Furthermore, we have previously shown that patients with poor prognostic markers would benefit from the early consolidative allo-HSCT after CAR T-cell therapy [28].

Many clinical trials showed that patients with high pre-infusion CD19-positive leukemia burden had low levels of anti-CD19 CAR T cells, increasing the probability of post-CAR T-cell therapy relapse [1, 28, 43]. The decrease in surface CAR expression may result from receptor internalization induced by exposure to CD19-positive leukemic cells [44]. Moreover, a high risk of relapse is observed in patients with high pre-infusion leukemia burden, even in those showing robust CAR T cell expansion. A high ratio of peak CAR T cell expansion to leukemia burden (> 1) was found to predict longer EFS and OS, suggesting that an optimal ratio of CAR T cells to CD19-positive leukemia cells is more probable in patients with a low leukemia burden than in those with a high leukemia burden, despite the lower CAR T cell expansion in patients with a low leukemia burden [1]. Intriguingly, we have previously shown that in patients with high leukemia burden (pre-infusion bone marrow MRD ≥5% as assessed by flow cytometry), consolidative allo-HSCT after CAR T-cell therapy significantly prolonged EFS and RFS [28].

Although high pre-lymphodepletion LDH level (> 210 U/L) and low platelet count (< 100,000/μL) showed little association with bone marrow leukemia burden, they were strongly correlated with the need of systemic bridging chemotherapy between leukapheresis and lymphodepletion [27]. Besides, high LDH levels and low platelet count were more frequent in patients with cytogenetic alterations and those with extramedullary diseases, respectively. Stepwise multivariate modeling revealed that normal pre-lymphodepletion LDH (≤210 U/L) and platelet count (≥100,000/μL) were independent predictors of prolonged EFS in patients exhibiting MRD-negative CR. Other studies also indicated that serum LDH levels were related to an immunosuppressive microenvironment, tumor burden, and proliferative activity in B-cell and other malignancies [45–50]. Hence, high pre-lymphodepletion LDH concentration and low pre-lymphodepletion platelet count can reflect not only leukemia burden, but also an aggressive disease phenotype, making them potential high-risk indicators of post-CAR T-cell relapse.

### Lymphodepletion regimen

Several clinical studies indicated that the incorporation of fludarabine to the lymphodepletion regimen could improve the expansion and persistence of CAR T cells, rate and depth of response, as well as OS and RFS, in r/r B-ALL and other B-cell malignancies [2, 3, 27, 31, 51]. Delay or abrogation of an immune response against the murine single-chain variable fragment component of the CAR, increased levels of cytokines that support T cell proliferation and survival, and IDO downregulation in tumor cells may contribute to the enhanced antitumor activity of CAR T cells after strong lymphodepletion [3, 51, 52]. Therefore, patients that did not receive fludarabine are at high risk of relapse and may require consolidative allo-HSCT to achieve sustained remission.

### Post-infusion monitoring

The depth of remission determined by marrow leukemic index clones for *IGH*, *IGK*, *TRB*, *TRD*, and *TRG* (detected by high throughput sequencing) has been associated with favorable long-term outcomes after CAR T-cell therapy [27]. Studies indicated that the absence of leukemic clones three weeks after CAR T-cell infusion in MRD-negative CR patients was associated with prolonged EFS and OS. Therefore, patients with persistent leukemic clones should be recommended for consolidative allo-HSCT to deepen remission and reestablish hematopoietic system and immune system.

A low peak and a short persistence of CAR T cells have also been associated with CD19-positive relapse, and most patients with CD19-positive relapse had either undetectable or very low (< 30 copies/μg DNA) CAR T cell counts before or at the time of relapse [27, 28]. Additionally, the persistence of functional CAR T cells for more than six months was a critical determinant of durable remission [32]. Furthermore, as the presence of BCA indicates sustained immunosurveillance of functional CAR T cells, a short duration of BCA (less than three months) was identified as a factor associated with an increased risk of CD19-positive relapse, and the median time from loss of BCA to CD19-positive relapse was 3.7 months [2, 20, 27]. Encouragingly, the MRD-negative CR patients who developed recovery of normal B cells within 63 days after CAR T cell infusion could obtain a certain benefit from consolidative allo-HSCT [23]. Therefore, the recovery of normal B cells can be regarded as an indicator of following CD19-positive relapse and can potentially provide a window for consolidative allo-HSCT.

Overall, relapse after CAR T-cell therapy can be potentially predicted by the aforementioned parameters, which exist during the whole procedure of CAR T-cell therapy, including screening, lymphodepletion, and follow-up. The prior existence or newly occurrence of these factors may facilitate timely treatment with consolidative allo-HSCT, improving disease outcomes.

## Therapeutic window and consolidative Allo-HSCT regimen

The median time for consolidative allo-HSCT in most centers ranged from 44 days to 84 days after CAR T-cell infusion (Table 1), before or immediately at the occurrence of high-risk factors. A long-term follow-up study indicated that late consolidative allo-HSCT (more than 80 days after CAR T-cell infusion) significantly increased the risk of death [33]. A recent published retrospective study indicated a lower incidence of relapse and a higher 2-year LFS in pre-transplant MRD-negative patients than in pre-transplant MRD-positive patients [53]. Besides, the relapse rate of MRD-negative CR patients was approximately 10% three months after CAR T-cell therapy [1, 21, 28]. These findings support the usefulness of early transplantation discussion and HLA typing for suitable B-ALL patients before the administration of CAR T-cell therapy, and we recommend treatment with consolidative allo-HSCT within three months of CAR T-cell therapy to maximize its benefit and minimize the risk of toxicity-related death.

As prior anti-CD19 CAR T-cell therapy can modulate the immune microenvironment and cause endothelial damage, the therapeutic protocol for consolidative allo-HSCT may need to be re-customized [54–56]. The donor types, graft cell types, conditioning therapies, and GVHD prophylaxis varied among several large-scale studies (Table 2). Overall, the protocols were similar to conventional allo-HSCT in r/r B-ALL. A recent study with a median follow-up of 36 months revealed no possible toxicity that was disproportionately more common among patients receiving consolidative allo-HSCT than in those undergoing allo-HSCT without prior CAR T-cell therapy [33]. Although there are no studies comparing different consolidative allo-HSCT protocols, available preliminary data suggest that conventional protocols are tolerable and feasible in most cases. Furthermore, researchers found that CAR T-cell therapy followed by CD34-selected T-cell depleted allo-HSCT was associated with lower incidence of TRM and prolonged OS when compared with unmodified allo-HSCT [57].

**Table 2:** Consolidative allo-HSCT regimen

Register number	Donor type	Cell type	Conditioning therapy	GVHD prophylaxis
ChiCTR-llh-16008711 [26]	17 Haploidentical 7 MUD 3 MSD	/	27 MAC (24 TBI/CY/Ara-C/MeCCNU/±ATG, 3 Bu/CY/Ara-C/MeCCNU/±ATG)	/
NCT01865617 [27]	3 MRD 9 MUD 1 mMURD 1 Haploidentical 5 UCT	13 PBSC 1 BM 5 Cord	14 MAC 2 RIC 3 NMA	5 CNI + MMF 1 CNI + MMF + sirolimus 9 CNI + MTX 3 CNI + MTX + abatacept 1 CNI + MMF + PtCy
NCT02965092 & NCT03366350 [28]	8 MSD 13 haploidentical	16 PBSC 5 PBSC+BM	21 MAC (VP16 + BUCY)	8 CsA/FK506 + MTX 13 FK506 + MTX + MMF + CD25 antibody+ATG
NCT03173417 [30]	16 MSD 9 MUD 50 Haploidentical	/	75 MAC (69 TBI-based for >5y patients, 6 Bu-based for ≤5y patients)	75 CsA + MTX + MMF

*Ara-C* cytarabine, *ATG* anti-thymocyte globulin, *BM* bone marrow, *Bu* Busulfan, *BUCY* busulfan and cyclophosphamide, *CNI* calcineurin inhibitor, *CsA* Cyclosporine, *CY* cyclophosphamide, *FK506* tacrolimus, *MAC* myeloablative conditioning, *MeCCNU* methyl-CCNU-semustine, *MMF* mycophenolate mofetil, *mMURD* mismatched unrelated donor, *MRD* matched related donor, *MSD* matched sibling donor, *MTX* methotrexate, *MUD* matched unrelated donor, *NMA* nonmyeloablative conditioning, *PBSC* peripheral blood stem cell, *PtCy* posttransplant cyclophosphamide, *RIC* reduced-intensity conditioning, *TBI* total body irradiation, *UCT* umbilical cord transplant, *VP16* etoposide

## Necessity of consolidative Allo-HSCT

Despite the potential benefit and safety of consolidative allo-HSCT after CAR T-cell therapy, not every patient should undergo allo-HSCT considering cost effectiveness and other potential risks. Factors, such as age, prior transplantation, CAR T-cell design, and treatment protocol should be taken into account in the decision making for consolidative allo-HSCT.

While adult r/r B-ALL patients typically benefit from consolidative allo-HSCT, pediatric and young adult patients may achieve long-term remission without consolidative allo-HSCT, with the 12-month EFS of approximately 50% when only a quarter patients were referred to allo-HSCT [2, 4]. Apart from differences in the mechanisms underlying leukemia development, the superior performance of pediatric patients might derive from a better quality of isolated T cells, as adult r/r B-ALL patients were probably heavily-treated [58].

Generally, patients undergoing allo-HSCT before CAR T-cell therapy are precluded from consolidative allo-HSCT, since second or third allo-HSCT has been associated with high risks of TRM [20, 27, 28]. Despite this, some patients still underwent second allo-HSCT after donor-derived CAR T-cell therapy. In the Sleeping Beauty-engineered donor-derived CAR T-cell therapy for B-ALL patients relapsed after allo-HSCT, 2 of the 6 MRD-negative CR patients received consolidative allo-HSCT and remained leukemia-free, the other 4 did not receive second allo-HSCT and 2 relapsed with CD19-positive disease [59]. In the SCRI-CAR19v1 study, 10 of 33 patients with a history of allo-HSCT received second allo-HSCT after CAR T-cell therapy-mediated CR, and five patients showed long-term remission [23]. Of the 23 patients who did not receive second allo-HSCT, eight patients showed long-term remission. Although the role of consolidative allo-HSCT was not clear in patients previously treated with allo-HSCT, these patients can still benefit when with short persistence of functional SCRI-CAR19v1. Therefore, a decision regarding a second allo-HSCT after donor-derived CAR T-cell therapy should be made on a case-by-case basis.

Preclinical studies indicated that CAR T cells with CD28 costimulatory domain had an increased expression of exhaustion-related genes, resulting in a relatively short duration after infusion, despite robust expansion and good efficacy at early time [60, 61]. Consistently, CD28-based anti-CD19 CAR T cells exhibited a shorter persistence than 4-1BB-based anti-CD19 CAR T cells [1, 18–21, 29, 62]. Nevertheless, treatment strategies and outcomes of CD28-based CAR T-cell therapies varied significantly among different studies. Most patients at the National Cancer Institute and Sheba Medical Center underwent consolidative allo-HSCT and exhibited improved survival, whereas only half of MRD-negative CR patients received consolidative allo-HSCT at the Memorial Sloan Kettering Cancer Center, and no clinical benefit was observed [1, 21, 29]. At present, there is insufficient evidence to conclude whether CD28-based CAR T-cell therapy should be consolidated with subsequent allo-HSCT.

As novel technologies came up, the necessity of consolidative allo-HSCT should be discussed extensively. In a humanized anti-CD19 CAR T-cell study, no survival benefit was observed in patients receiving consolidative allo-HSCT [63]. Similarly, CAR designs or treatment strategies to reduce CD19-negative relapse or prolong the functional persistence of CAR T cells, such as sequential infusion of anti-CD19 and anti-CD22 CAR T cells and combination with PD-1 inhibitors, may provide long-term remission without consolidative allo-HSCT [64–68]. In particular, two patients received universal CAR T cells and all received consolidative allo-HSCT [69]. It raises the question of whether consolidative allo-HSCT is necessary after universal CAR T-cell therapy, as the CAR T cells would be eventually eliminated by immunological rejection [70].

Considering the high heterogeneity among and within patients, different CAR T cell products and continuous improved treatment strategies, the application of consolidative allo-HSCT cannot be simply generalized. However, consolidative allo-HSCT is strongly recommended for adult high-risk r/r B-ALL patients who have no history of allo-HSCT and achieve MRD-negative CR after murine anti-CD19 CAR T-cell therapy.

## Recommendation for the assessment and management of r/r B-ALL patients in CAR T-cell therapy

**Fig. 1:**
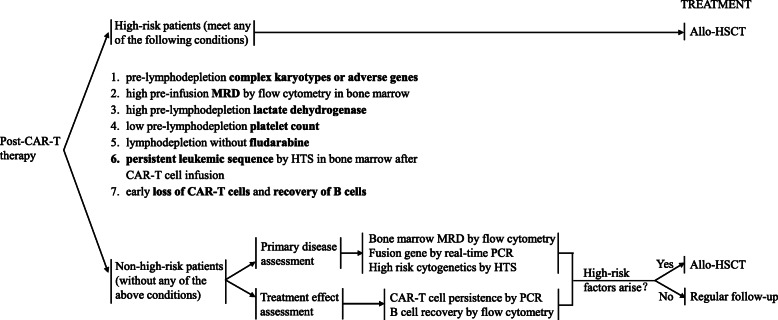
Recommendation for the assessment and management of r/r B-ALL patients in anti-CD19 CAR T-cell therapy. MRD, minimal residual disease; HTS, high throughput sequencing; PCR, polymerase chain reaction

Based on the findings of clinical trials and our institutional experiences, we established a recommendation for the assessment and management of r/r B-ALL patients undergoing anti-CD19 CAR T-cell therapy. (Fig. 1) High-risk patients should be considered for allo-HSCT, and suitable donors should be identified before CAR T-cell therapy, enabling the timely consolidative allo-HSCT if eligible. Patients without risk factors may achieve long-term remission, with continuous expression of CAR-targeted tumor antigen and durable functional CAR T cells [71]. Therefore, regular assessment on primary disease, including bone marrow MRD, fusion genes and high risk cytogenetics, and treatment effect, including CAR T cell persistence and B cell recovery, would enable the prompt identification of patients at a high risk of relapse. Rise of MRD, occurrence of high-risk genes or chromosome mutations, decrease of CAR T cells, and recovery of normal B cells, can be used as indications for consolidative allo-HSCT. However, there are still limitations of this recommendation, as no cut-off value but a trend was given for high-risk factors. More clinical data are needed to determine the optimal cut-off values.

## Conclusions

Consolidative allo-HSCT has been used in r/r B-ALL patients after anti-CD19 CAR T-cell therapy in several clinical trials and showed acceptable safety and efficacy. In addition to anti-CD19 CAR T-cell therapy, anti-CD22 CAR T-cell therapy could also be consolidated with subsequent allo-HSCT, providing favorable EFS, LFS, and OS, as well as exhibiting a satisfactory safety profile [72–74]. Consolidative allo-HSCT could also be applied in patients with other B-cell malignancies, including B-cell non-Hodgkin lymphoma and chronic lymphocytic leukemia [33]. These clinical data indicate a demand for discussion of consolidative strategy in more other targeted CAR T therapies and other diseases.

Based on available clinical data, we recommend consolidative allo-HSCT in eligible high-risk patients to reduce the incidence of recurrence after CAR T-cell therapy and improve the quality of life. However, future randomized clinical trials and long-term outcome data are required to establish the clinical value of allo-HSCT after CAR T-cell therapy. The role of consolidative allo-HSCT should be constantly redefined with the development of novel CAR designs and combination therapies.

## Data Availability

Data sharing is not applicable to this article as no datasets were generated or analyzed during the current study.

## Abbreviations

Allo-HSCT: Allogeneic hematopoietic stem cell transplantation
BCA: B-cell aplasia
CAR T: Chimeric antigen receptor-modified T cell
CR: Complete remission
CRm: Complete molecular remissions
cy: Cyclophosphamide
EFS: Event-free survival
EMD: Extramedullary disease
flu: Fludarabine
GVHD: Graft-versus-host disease
HTS: High throughput sequencing
LDH: Lactate dehydrogenase
LFS: Leukemia-free survival
MRD: Minimal residual disease
OS: Overall survival
RFS: Relapse-free survival
r/r B-ALL: Relapsed/refractory B-cell acute lymphoblastic leukemia
TRM: Treatment-related mortalities
